# The Effect of Contemporary Brachytherapy Practices on Prognosis in Women with Locally Advanced Cervical Cancer

**DOI:** 10.3390/curroncol30040326

**Published:** 2023-04-19

**Authors:** Janna J. Laan, Luc R. C. W. van Lonkhuijzen, Jaap A. Stokking, Danique L. J. Barten, Karel A. Hinnen, Bradley R. Pieters, Lukas J. A. Stalpers, Henrike Westerveld

**Affiliations:** 1 Department of Radiation Oncology, Amsterdam UMC Location University of Amsterdam, Meibergdreef 9, 1105 AZ Amsterdam, The Netherlands; 2 Cancer Center Amsterdam, Cancer Treatment and Quality of Life, 1105 AZ Amsterdam, The Netherlands; 3 Center for Gynaecologic Oncology, Amsterdam UMC Location University of Amsterdam, Meibergdreef 9, 1105 AZ Amsterdam, The Netherlands; 4 Department of Radiotherapy, Erasmus Medical Cancer Institute, University Medical Center Rotterdam, 3015 GD Rotterdam, The Netherlands

**Keywords:** cervical cancer, brachytherapy, prognosis, toxicity

## Abstract

(1) Background: Over the past two decades use of new imaging modalities and the adaptation of applicators have allowed for advances in volumetric (3D) imaging-based brachytherapy practices for patients with locally advanced cervical cancer. The aim of this study was to compare the oncological outcome and toxicity for three consecutively introduced brachytherapy practices in a large single-center cohort; (2) Methods: Patients treated for cervical cancer with primary radiotherapy and curative intent were consecutively included in this retrospective, single-center cohort study from 2006 to 2019. This cohort was divided into three groups (CT, MRI, and MRI+needles) based on the timing of the introduction of a novel brachytherapy practice; 3D brachytherapy planning using CT- and MRI-guided adaptive brachytherapy and the use of parametrial interstitial needles, respectively. Actuarial estimates were compared between groups. Multivariable Cox regression analyses were performed to correct for other risk factors. Crude rates of severe (≥grade 3) late toxicity were reported; (3) Results: A total of 397 patients were included in this cohort. At a median follow-up of 40 months (interquartile range (IQR) 22–62), actuarial 3-year local control, pelvic control, disease-free survival, and overall survival for the entire cohort were 91% (95% (Confidence Interval (CI)) 88–94), 88% (95% CI 84–91), 69% (95% CI 64–74), and 75% (95% CI 70–79), respectively). Local control, disease-free survival, and overall survival were significantly improved in the MRI+needles group compared to the CT group (*p* = 0.040, *p* = 0.004, and *p* < 0.001, respectively). Independent risk factors for overall survival were treatment in either the CT or MRI group (vs. MRI+needles), older age at diagnosis, adeno (squamous) carcinoma, FIGO stage III/IV, and lymph node metastases. The crude rate of severe late toxicity was 27% in the CT, 26% in the MRI, and 20% in the MRI+needles group; (4) Conclusions: Prognosis in women with locally advanced cervical cancer treated with state-of-the-art MRI-guided adaptive brachytherapy combined with parametrial interstitial needles compares favorably to patients treated with more traditional CT only based brachytherapy.

## 1. Introduction

Brachytherapy (BT) is an integral part of the treatment of locally advanced cervical cancer (LACC). The current standard of care for these women is external beam radiotherapy (EBRT) with concurrent chemotherapy (in general cisplatin) followed by brachytherapy [1,2]. Over the past two decades, several new BT practices have been introduced based on new imaging modalities and the adaptation of applicators.

Until two decades ago, brachytherapy was mainly based on radiographic imaging, which could not identify the target and organs at risk, nor the accuracy of applicator placement. In the early 21st century, 3D-based volumetric radiotherapy planning based on computed tomography (CT) was introduced, allowing dose optimization by visualizing organs at risk and applicator position. A standard treatment plan was constructed based on point dosimetry (generally to Manchester point A) [3]. Magnetic resonance imaging (MRI) provides better visualization of the tumor compared to computed tomography (CT) due to its superior soft tissue contrast. MRI-guided brachytherapy planning based on a tailored pear-shaped target volume instead of prescribed to a dose point has been increasingly used in daily practice following the publication of the recommendations of the GYNaecological working group of the Groupe Européen de Curiethérapie/European Society for Radiotherapy and Oncology (GYN GEC-ESTRO) describing the target volume concept based on MRI results and introducing a uniform dose reporting [4,5]. This resulted in both an improved coverage of the clinical target volume (CTV), including the visible tumor and adjacent presumed substantial tumor spread, and reduced doses to organs at risk (OAR) [6]. In general, brachytherapy consists of 1–3 applications. Image-guided adaptive brachytherapy (IGABT) consists of repeated 3D-imaging, preferably using MRI, for each session, which allows the dose to be adjusted according to changes in the patient’s anatomy (for example, tumor shrinkage, organ motion, and changes in applicator position) [7]. More recently, new commercially available applicators have introduced the possibility of inserting interstitial needles into the parametrium. This has allowed better coverage of target volumes, especially in larger tumors [8,9]. In patients with parametrial tumor extension, the use of interstitial needles improved local control (LC) [10]. These emerging BT practices have allowed for dose escalation without increasing the dose to the OARs.

The potential benefit of IGABT over 2D brachytherapy in patients with locally advanced cervical cancer has been evaluated in a few non-randomized studies. Patients treated with 3D-BT using CT had significantly better local control compared to a historical cohort treated with 2D-BT [11,12]. However, in patients with large tumors (>5 cm), pelvic control was only 75% at 3 years [11]. In 2007, Pötter et.al. published the first comparison between CT- and MRI-guided adaptive brachytherapy (MRI-IGABT). They found a 3-year LC rate of 85% for the entire cohort; however, they found no improvement in LC for patients with large tumors [13]. After the introduction of interstitial needles in the same center, 3-year local control increased to 92% for large tumors [14].

The studies that have compared different brachytherapy practices to date are relatively small or multicenter studies with wide variation in care practice and case mix. Therefore, our aim was to evaluate the clinical outcome of all three emerging brachytherapy practices in a large single-center cohort.

## 2. Materials and Methods

All patients treated with primary radiotherapy with curative intent for newly diagnosed and biopsy-proven cervical cancer treated at our center between October 2006 to April 2019 were included. Patients had to have received at least EBRT at our center in order to collect sufficient data on EBRT and follow-up. Patients scheduled for curative radiotherapy who were unable to complete radiotherapy for any reason and therefore did not receive a curative dose were excluded. Patients who were lost to follow-up during or in the first 2 weeks after treatment were also excluded from the analysis. In general, patients treated with primary radiotherapy at our center were followed up every three months for the first two years after treatment and, thereafter, every 6 months for up to 5 years after treatment.

Patient, tumor, and treatment characteristics, disease status, survival, and severe late toxicity were collected retrospectively from the patient records. All patients were clinically staged according to the 2009 International Federation of Gynaecology and Obstetrics (FIGO) criteria and the TNM Classification of Malignant Tumors [15]. Treatment consisted of EBRT, usually combined with weekly cisplatin 40 mg/m^2^ (or locoregional hyperthermia if contraindicated), followed by a BT boost to the cervix. Pelvic EBRT was delivered in 23–28 fractions of 1.8–2.0 Gy. The clinical target volume for pelvic EBRT was extended to the para-aortic region in the presence of pathological lymph nodes in the common iliac or para-aortic regions. If the lower third of the vagina was involved (none of the patients had pathological inguinal lymph nodes), radiation to the groins was indicated. Age (as a continuous variable) was assessed as the age at the time of pathological diagnosis. Performance score was assessed according to the WHO classification as described in the medical record at the first consultation (at baseline) [16]. Patients with a WHO performance score of 0 were compared with patients with a higher (worse) performance score. Patients who had never smoked were compared with patients who had (recently) quit smoking or who were active smokers at the time of the first consultation (current smokers). Malnutrition was defined as a BMI < 18.5 kg/m^2^ (18–69 years) or <20 kg/m^2^ (>70 years) [17]. Both hypertension and diabetes mellitus were assessed based on medical history and medication use at the first consultation. Detection of lymph node metastases was mainly based on imaging and, in some cases, on a pathological sample. More conformal EBRT techniques (box and 3D-CRT) were assessed separately from newer techniques (IMRT and VMAT), which may allow for better OAR sparing and, therefore, reduce toxicity. Overall treatment time was defined as the time from the start of EBRT to the day after the last application of brachytherapy. Based on previous literature describing a worse prognosis in patients treated longer than 49–56 days, total treatment time was dichotomized with >50 days as the cut-off [18,19]. After data collection, patients were divided into three subgroups. These groups were defined on the basis of treatment, which approximated the date of introduction of a new brachytherapy practice. In all three groups, brachytherapy was typically delivered in 1–2 applications using a tandem-ovoid applicator and pulsed dose rate (PDR).

The CT group consisted of all pati”nts ’reated between October 2006 and September 2009. During this time, organs at risk were contoured, and doses were registered; however, a standard BT plan with a dose prescription of 24 × 1 Gy at point A was used [3].

The MRI group consisted of all patients treated between September 2009 and January 2013. MRI-guided brachytherapy was introduced at our center in 2009. Since then, target delineation and dose reporting have been performed according to the newly introduced GYN GEC-ESTRO guidelines [4,5]. The dose was prescribed to the clinical target volume (more explicitly to the D90 of the high-risk CTV). An EBRT boost was given to a summated total dose of 60 Gy in EQD2_α/β10_ (equivalent dose in 2Gy fractions) in case of parametrial involvement.

The MRI+needles group consisted of all patients treated after January 2013. During this period, our center widely adopted the use of parametrial interstitial needles during MRI-guided adaptive brachytherapy. The use of these interstitial needles was optional but was always indicated in case of expected suboptimal target coverage (for example, in case of parametrial involvement) or to better spare the OAR compared to the intracavitary-only technique. Thus, the MRI+needles group represents patients with access to state-of-the-art MRI-guided adaptive brachytherapy with combined intracavitary/interstitial technique.

Our center gradually applied dose escalation aiming at 80–85 Gy in the early years and 90–95 Gy to the clinical target volume in recent years. From around 2015, a simultaneous (instead of sequential) EBRT boost to a total dose of 60 Gy in EQD2 _α/β10_ was applied to pathological lymph nodes. From 2008, tumor staging included an FDG PET-CT scan (in addition to an MRI).

Predefined clinical endpoints included local control (LC), pelvic control (PC), disease-free survival (DFS), and overall survival (OS). All clinical endpoints were assessed by the time in months from pathological diagnosis to recurrence or death. Recurrence was defined as local if in the cervix, parametria, uterine corpus and/or vagina, and pelvic if within the EBRT field. Patients treated with extended-field radiotherapy and para-aortic lymph node metastases that were in-field were classified as pelvic recurrence. Out-field para-aortic lymph node metastases were classified as distant recurrence. Disease-free survival was defined as the time from pathological diagnosis to any recurrence. Overall survival was defined as the time from pathological diagnosis to death from any cause.

Severe late toxicity was defined as grade 3 or higher according to the Common Terminology Criteria for Adverse Events (CTCAE) v4.03 scale and occurring at least 3 months after treatment [20].

Descriptive statistics for all characteristics collected were reported as means or medians with interquartile ranges (IQRs) for continuous and percentages for categorical variables. Toxicity was assessed by both the crude incidence of the highest grade per patient and the incidence of each type of severe toxicity (per event). Patients were censored for toxicity at the time of any recurrence. Fisher-Freeman-Halton analysis for binary variables, Kruskal-Wallis analysis for continuous non-normally distributed variables, one-way ANOVA analysis for continuous normally distributed variables, and Mantel-Haenszel test for trends in ordinal variables were used to compare baseline patient and treatment characteristics. Actuarial estimates and graphical analyses for the distribution of LC, PC, DFS, and OS were performed using the Kaplan-Meier method. The log-rank test was used to compare outcomes between groups. Patients without an event were censored at the last date of consultation. If the overall log-rank test was statistically significant, pairwise post hoc tests were performed. To assess patterns of recurrence, a Venn diagram was constructed for all patients and stratified by the FIGO stage.

Cox regression analyses were performed to identify risk factors for LC, PC, DFS, and OS. For each clinical outcome, a predefined set of variables was selected for univariable analysis based on a consensus meeting involving both a radiation oncologist and a gynecologist. Multivariable analysis was performed using the backward conditional method for all variables with a *p*-value less than 0.1 (Akaike Information Criterion). Multivariable analysis was performed only for outcomes with ≥50 events.

For all statistics, a probability (*p*-) value of 0.05 or less was considered significant. Baseline characteristics were compared, and a Cox regression analysis was performed using IBM SPSS statistics software (version 26). Survival analysis was performed in R (version 4.0.3) using the ‘survival’, ‘survminer’, and ‘ggplot2’ packages.

## 3. Results

### 3.1. Patient Characteristics

**Figure 1:**
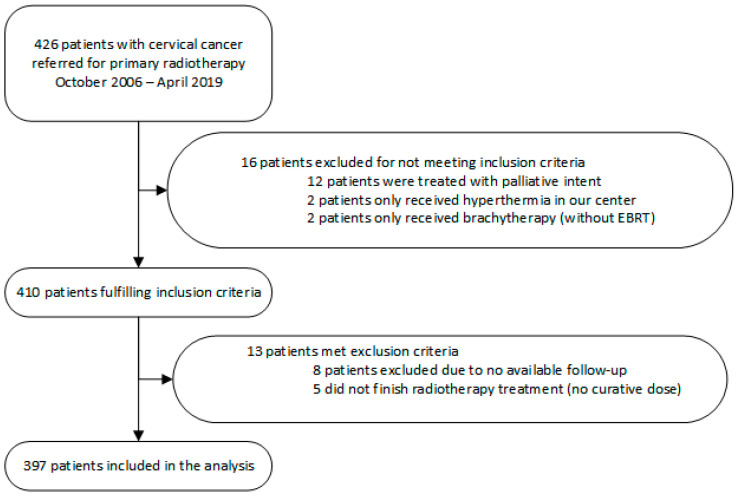
Patient inclusion and exclusion flowchart.

A total of 426 patients with primary cervical cancer were referred to our outpatient clinic for primary radiotherapy between October 2006 and April 2019. A total of 397 patients were included in the analyses. The reasons for exclusion are shown in Figure 1.

The CT, MRI, and MRI+needles groups consisted of 75, 103, and 219 patients, respectively. Baseline patient and treatment characteristics by treatment group are shown in Table 1. The median age was 54 years (IQR 42–70). The median follow-up was 40 months (IQR 22–62). Almost all patients had an MRI as part of their diagnostic work-up (366 patients, 92%), and 302 (76%) also had a PET-CT scan. Parametrial interstitial needles were used in 156 of the 219 patients (71%) in the MRI+needles group.

### 3.2. Survival

**Figure 2:**
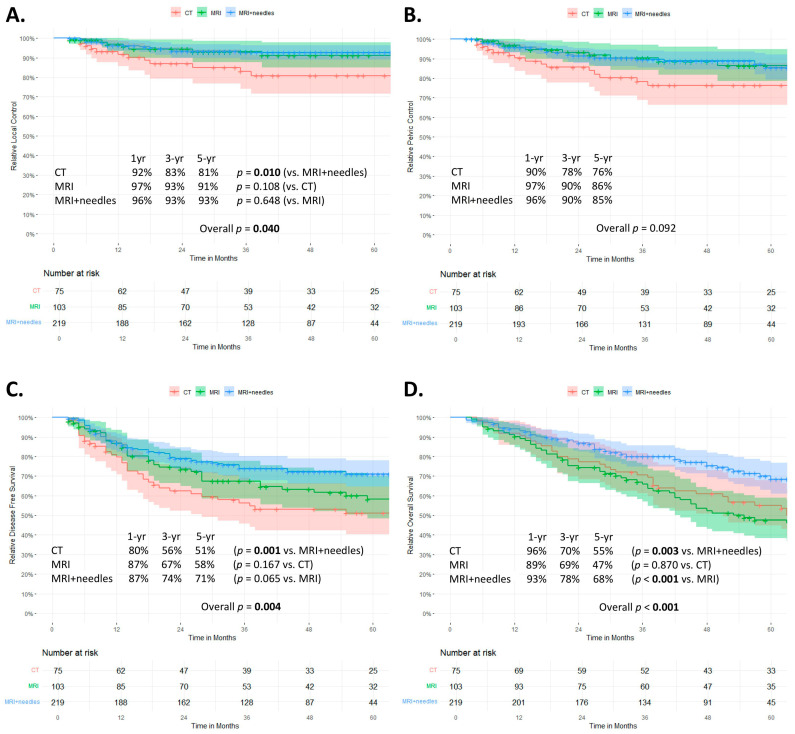
Kaplan-Meier probability estimates stratified by treatment group for (**A**) local control, (**B**) pelvic control, (**C**) disease-free survival, and (**D**) overall survival.

Tumor control and survival curves using Kaplan-Meier probability estimates stratified by treatment subgroup are shown in Figure 2. At 3 and 5 years, the LC rates were 91% (95% CI 88–94) and 90% (95% CI 87–93), the PC 88% (84–91) and 84% (80–88), the DFS 69% (64–74) and 63% (58–69), and the OS 75% (70–79) and 60% (55–66), respectively.

Local control rates and DFS were significantly worse in the CT group compared to the MRI+needles group (*p* = 0.040 and *p* = 0.004, respectively) (Figure 2A,C). The overall log-rank test showed no significant differences in pelvic control between the three groups. Although no further post hoc testing was performed, there was a trend towards a lower pelvic control rate in the CT group compared to the more recent cohorts (Figure 2B). Overall survival was significantly better in the MRI+needles group compared to both the CT (*p* = 0.003) and MRI groups (*p* < 0.001) (Figure 2D).

The 3-year local control rate for patients with FIGO stage I/II was 86%, 97%, and 95% in the CT, MRI, and MRI+needles group, respectively. For FIGO stage III/IV, the 3-year local control rate was 79%, 88%, and 86%, respectively (Supplementary Figure S1A).

The actuarial OS for FIGO I/II at 3 years was 78%, 72%, and 86% for the CT, MRI, and MRI+needles groups, respectively. For FIGO III/IV, the 3-year actuarial OS was 56%, 58%, and 63% for the CT, MRI, and MRI+needles groups, respectively (Supplementary Figure S1B).

**Figure 3:**
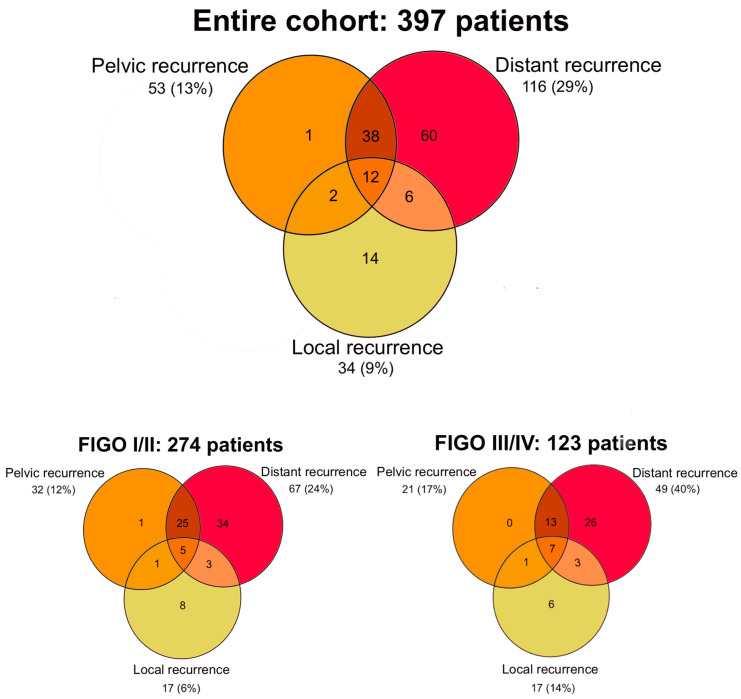
Venn diagrams of local, pelvic, and distant recurrence rates for the entire cohort and stratified by FIGO_2009_ stage.

Figure 3 shows the Venn diagrams for local, pelvic (in-field), and distant (out-field) recurrences for the entire cohort and stratified by FIGO stage. Most recurrences were either solitary distant recurrence (60/397, 15%) or both pelvic and distant recurrence with or without local failure (50/397, 13%). Para-aortic recurrences could occur inside the radiation field (pelvic recurrence) or outside the radiation field (distant recurrence). In total, 30 (8%) of the 397 patients developed a para-aortic recurrence. Of these, 8 were pelvic recurrences, and 22 were distant recurrences. Of the 60 patients with a solitary distant recurrence, six (10%) patients had a solitary para-aortic lymph node recurrence. Only 14 patients (4%) of the entire cohort developed an isolated local recurrence. Of these, 4/75 were in the CT group, 4/103 in the MRI group, and 6/219 in the MRI+needles group.

### 3.3. Risk Factors

Table 2 presents the results of univariable and multivariable Cox Regression analysis for local control, pelvic control, disease-free survival, and overall survival.

Of the 397 women, 133 developed a recurrence. At the end of the follow-up, 159 women had died. Of these 159, 97 died of cervical cancer recurrence, 4 died of other malignancies, 19 died of other causes, and 36 died of unknown causes.

In univariable Cox Regression analyses, risk factors for worse local control were hypertension, FIGO stage III/IV, adeno (squamous) carcinoma, and treatment in the CT group (compared with the MRI+needles group). Significant risk factors for worse disease-free survival in both uni- and multivariable analysis were as follows: high FIGO stage, adeno (squamous) carcinoma, lymph node involvement, and treatment in the CT group (compared to the MRI+needles group). In univariable Cox regression analyses for overall survival, patients diagnosed at an older age, with hypertension, FIGO stage III/IV, adeno (squamous) carcinoma, and treatment received in the CT or MRI groups had a significantly increased risk. In multivariable analysis, patients treated in the MRI+needles group had significantly improved survival even after correction for these other risk factors. In addition, older age at diagnosis, FIGO stage III/IV, adeno (squamous) carcinoma, and lymph node metastases were significant independent risk factors for overall survival.

### 3.4. Toxicity

There were 110 severe late toxicity events in 91 patients (crude rate 23%; 91/397). Of them, 41 (10%) patients developed severe gastrointestinal toxicity, 38 (10%) patients developed severe vaginal toxicity, and 19 (5%) patients developed severe genitourinary toxicity. Other severe toxicity occurred in 12 (3%) patients. Seven had insufficiency fractures requiring intervention, three had severe abdominal pain requiring surgery, one had radiation-induced vascular disease, and one reported fatigue that limited self-care. Of all cases of vaginal toxicity, 80% were due to vaginal stenosis interfering with sexual activity or gynecological examination. Thirteen patients (3%) developed multiple types of severe toxicities. Two patients died as a result of toxicity (grade 5). The causes of death were sepsis due to rectovaginal fistula and complications from radiation-induced vasculopathy. The median time from the end of treatment to the onset of severe toxicity was 12 months (IQR 6–28 months). Table 3 shows the crude rate of severe toxicity per treatment group.

After excluding severe vaginal stenosis, 18 of the 75 patients in the CT group developed severe late toxicity (24%), compared to 24 of 103 (23%) in the MRI group and 32 out of 219 (14%) in the MRI+needles group (*p* = 0.002). The rate of severe late toxicity was significantly higher in patients treated with older external beam radiation techniques (3DCRT/Box technique) compared to the more recently introduced IMRT/VMAT (30% vs. 20%) *(p* = 0.031).

## 4. Discussion

In our cohort, patients treated for cervical cancer in the group with the most conventional brachytherapy planning using only CT had an increased risk of (local) recurrence and death compared with the group treated with state-of-the-art MRI-IGABT with interstitial needles. This difference was independent of other known risk factors, such as histology, performance status, age, and tumor stage. Simultaneously, patients treated in the latest cohort had the lowest rate of severe toxicity, despite the use of dose escalation.

The excellent local control rates of the patients treated with MRI-IGABT are similar to previous reports [21]. Although the multi-institutional retroEMBRACE reported on the impact of MRI-IGABT on oncological outcomes and survival in a larger number of patients, they did not directly compare the different modalities used for brachytherapy [22]. The 581 patients treated with MRI-IGABT in the retroEMBRACE had a 3-year LC of 85–95% (depending on tumor size). Our results are comparable with an overall 3-year LC of 93% in the MRI group. One of the main conclusions from the retroEMBRACE was that IGABT improved LC by ~10% compared to conventional 2D brachytherapy, which was also confirmed by our results (with LC rates of 83% and 93% in the CT and MRI groups, respectively). The significantly improved LC after the introduction of interstitial needles may be due to the ability to escalate the clinical target volume dose and improve the coverage of larger tumors [10]. The 3-year pelvic (in-field) control rate of 88% in the entire cohort compares favorably with other studies (70–85%) [12,22,23]. Although these studies did not report the use of PET-CT, most of the patients included in these studies were treated at a time when PET-CT was not yet standard of care. Women in our MRI+needles group have a comparable 3-year PC rate (90%) to the first publication with MRI-IGABT, including the use of interstitial needles (91%) [14]. These excellent local and pelvic control rates are supported by the low rate of solitary in-field recurrences (local +/− pelvic) (16 of 397 women). Moreover, solitary out-field para-aortic lymph node metastases occurred in only six women.

Disease-free survival improved in each consecutive cohort, with only the difference between the CT and MRI+needles groups reaching statistical significance. The use of PET-CT was introduced in 2008 and became the standard of care for the initial assessment of disease extent in the following years. About half of the patients in the CT group, three-quarters of the patients in the MRI group, and almost all of the patients in the MRI+needles group received PET-CT. This may have resulted in stage migration due to improved imaging of nodal involvement, leading to an extended EBRT field or primary chemoradiation instead of surgery [24]. In the current cohort, patients treated in the CT group had a worse DFS. This significant difference remained after correction for nodal status (and FIGO stage); however, only 45% of patients in the CT group received a PET-CT, and therefore, understaging cannot be excluded.

Overall survival was significantly better in the MRI+needles group compared with the other two groups. Rijkmans et al. found that overall survival was significantly improved in patients treated with MRI-IGABT compared with conventional CT brachytherapy planning, independent of age, tumor size, FIGO stage, and chemotherapy [25]. Although we also found that treatment in the MRI+needles group improved survival independently of other risk factors, we did not see an improvement in survival in the MRI group compared with the CT group. However, there were significant differences between the three groups for hypertension and overall treatment time, which may have been unfavorable for the MRI group. Contrarily, more women in the MRI+needles group were (former) smokers than in the MRI group.

Previously, retroEMBRACE and EMBRACE-I have reported toxicity rates in this patient population. Unfortunately, the comparison of different cohorts remains challenging due to reporting bias and differences in case mix. RetroEMBRACE is the largest retrospective cohort of patients treated for cervical cancer with primary radiotherapy according to the GEC-ESTRO recommendations (using CT/MRI-based brachytherapy) [22]. The crude rate of gastrointestinal and genitourinary severe late toxicity events combined was 8% (58/731) in the retroEMBRACE and 15% (60/397) in our cohort [22]. However, most patients in the MRI+needles group are treated according to the GEC-ESTRO recommendations, and in this group, the combined crude gastrointestinal and genitourinary toxicity rate was 11%. The prospective EMBRACE-I reported a crude severe toxicity rate of 16% (195/1251) compared to 20% (44/219 in the MRI+needles group) in our cohort [26].

Other studies report a wide range (2–21%) of crude rates of severe late toxicity [12,25,27]. Notably, these studies mainly reported toxicity according to CTCAE v3.0, whereas we report toxicity grade according to CTCAE v4.03. This may influence the reported toxicity rate. For example, grade 3 vaginal stenosis in CTCAE v4.03 is defined as interference with sexual activity or gynecological examination, whereas in CTCAE v3.0, grade 3 was defined as complete obliteration, which is rarely seen [28]. There were no significant differences between the three groups when comparing the crude rate of all types of severe late toxicities combined. However, when vaginal toxicity was excluded from this comparison, there was a significant reduction in the rate of severe toxicity in the MRI+needles group, despite the use of dose escalation in this group.

Factors other than brachytherapy practice that might influence radiotherapy toxicity, such as EBRT technique, dose escalation, and method of toxicity reporting, changed during the inclusion period. New EBRT techniques (IMRT/VMAT) are designed to better spare the OAR without changing the dose to the tumor. All patients in the MRI+needles group and only one in the CT group were treated with IMRT/VMAT. Previous studies highlight the reduced toxicity rate after IMRT or VMAT compared to 3D-CRT, and our results confirm this finding [29,30]. These studies found similar oncological outcomes among patients treated with 3D-CRT and IMRT/VMAT. Therefore, it is unlikely that the improved prognosis in the MRI+needles group reflects the change in the EBRT technique.

To our knowledge, this is one of the largest cohorts comparing oncological outcomes and survival between three 3D-brachytherapy practices. The present study is the first to correct for several potential confounders in a multivariable model with three brachytherapy practices, including state-of-the-art IGABT with the option of interstitial needle insertion. One of the limitations of this study is that toxicity was assessed retrospectively based on the medical records in the early years, whereas from 2017 onwards, a mandatory toxicity assessment was introduced in our electronic patient database at each follow-up visit. This may have resulted in less under-reporting in the MRI+needles group as it was scored prospectively. Interestingly, the rate of severe toxicity did not increase as a result of this mandatory scoring system; in fact, genitourinary toxicity decreased considerably. We recognize the other limitations of a retrospective cohort study. Most importantly, other staging and treatment modalities changed simultaneously with brachytherapy practice. Another limitation of our study is that we were not able to report on the influence of the brachytherapy dose on the outcome. This is due to the fact that CT cannot be used to delineate a clinical target volume according to the GEC-ESTRO recommendations (contouring recommendations are based on MRI results because of their superior soft-tissue contrast). Since our center’s participation in the EMBRACE-II study, a gradual dose escalation has been applied with an expected improvement in a 3-year LC of 1–4% [31].

## 5. Conclusions

In this large single-center study, MRI-IGABT combined with parametrial interstitial needles leads to excellent tumor control (3-year LC, PC, and DFS of 93%, 90%, and 74%, respectively) and survival (3-year OS of 78%) without an increased risk of severe late toxicity compared to more conventional brachytherapy practice.

## Figures and Tables

**Table 1:** Baseline patient and treatment characteristics per treatment group.

Variable		CT		MRI		MRI+Needles		*p*-Value
		n = 75	(%) IQR	n = 103	(%) IQR	n = 219	(%) IQR	
Age ^1^	Median	55	42–71	60	44–72	53	41–67	0.136
WHO Performance score	0	43	(57)	58	(56)	142	(65)	0.254
	≥1	32	(43)	45	(44)	77	(35)	
Smoking	Smoker *	36 ^a,b^	(48)	47 ^b^	(46)	128 ^a^	(58)	**0.023**
	never smoked	38 ^a,b^	(51)	56 ^b^	(54)	83 ^a^	(38)	
	missing	1	(1)	0	(0)	8	(11)	
Nutritional status	malnourished	8	(10)	8	(8)	15	(7)	0.209
	not malnourished	47	(63)	79	(77)	196	(89)	
	missing	20	(27)	16	(15)	8	(4)	
Hypertension	yes	20 ^a,b^	(27)	36 ^b^	(35)	44 ^a^	(20)	**0.016**
	no	55 ^a,b^	(73)	67 ^b^	(65)	175 ^a^	(80)	
Diabetes Mellitus	yes	6	(8)	9	(9)	15	(7)	0.842
	no	69	(92)	94	(91)	204	(93)	
FIGO stage ^2^	I	18 ^a^	(24)	19 ^a^	(18)	62 ^a^	(28)	**0.043**
	II	32 ^a^	(43)	43 ^a^	(42)	100 ^a^	(46)	
	III	18 ^a^	(24)	29 ^a^	(28)	46 ^a^	(21)	
	IV	7 ^a,b^	(9)	12 ^b^	(12)	11 ^a^	(5)	
Histology	SCC	67	(89)	88	(85)	185	(85)	0.612
	Adeno(squamous)	8	(11)	15	(15)	34	(15)	
Nodal status	N1	37	(49)	46	(45)	115	(52)	0.417
	N0	38	(51)	57	(55)	104	(48)	
PET-CT scan	yes	34 ^a^	(45)	76 ^b^	(74)	189 ^c^	(86)	**<0.001**
	no	41 ^a^	(55)	27 ^b^	(26)	30 ^c^	(14)	
EBRT technique	Box/3D-CRT	62 ^a^	(83)	66 ^b^	(64)	2 ^c^	(1)	**<0.001**
	IMRT/VMAT	1 ^a^	(1)	22 ^b^	(21)	212 ^c^	(97)	
	missing	12	(16)	15	(15)	5	(2)	
Chemotherapy	yes	56	(75)	74	(72)	154	(70)	0.798
	no	19	(25)	29	(28)	65	(30)	
Hyperthermia	yes	16	(21)	22	(21)	45	(20)	0.971
	no	59	(79)	81	(79)	174	(80)	
Overall Treatment Time ^3^	>50 days	10 ^a^	(13)	27 ^b^	(26)	27 ^a^	(12)	**0.008**
	Median	44	39–48	46	43–51	45	41–49	**0.027**

^a,b,c^ Groups with the same subscript (i.e. both ‘a’) are not significantly different. Groups with different subscripts (i.e. ‘a’ and ‘b’) are significantly different. Q1; Q3 = interquartile range; SCC = squamous cell carcinoma; N1 = with lymph node metastases; N0 = no lymph node metastases; 3D-CRT = three-dimensional conformal radiation therapy; IMRT = intensity-modulated radiotherapy; VMAT = volumetric-modulated arc therapy. Legend: * current or former smoker at diagnosis, ^1^ age at diagnosis in years (continuous) evaluated by one-way ANOVA; ^2^ Mantel-Haenszel test; ^3^ Time in days from the start of EBRT to the day after the last application of brachytherapy evaluated by Kruskal-Wallis Test; all other variables are analyzed by Fisher-Freeman-Halton exact tests. Bold = *p*-value < 0.05. Reported *p*-values correspond to the probability of a three-way comparison.

**Table 2:** Univariable and multivariable Cox regression analysis for local control, pelvic control, disease-free survival, and overall survival.

	Univariable		Multivariable	
Local Control	*p*-Value	HR (95% CI)	*p*-Value	HR (95% CI)
Smoking: current or former smoker (v never smoked)	0.800	0.92 (0.47–1.80)		
Nutritional status: malnourished at baseline (v not malnourished at baseline)	0.298	0.04 (0.00–15.90)		
Hypertension (v no hypertension)	0.004	2.69 (1.36–5.31)	NA	
Diabetes mellitus (v no diabetes mellitus)	0.744	1.22 (0.37–3.99)		
FIGO stage III/IV (v stage I/II)	0.004	2.71 (1.38–5.32)	NA	
Histology: adeno (squamous) (v squamous cell)	0.032	2.30 (0.07–4.93)	NA	
Overall treatment time ^1^: >50 (v ≤ 50 days)	0.397	1.43 (0.62–3.30)	NA	
Treatment subgroup (v MRI+needles)	0.049		NA	
CT	0.017	2.57 (1.19–5.58)	NA	
MRI	0.609	1.26 (0.53–3.00)		
Pelvic control				
Smoking: current or former smoker (v never smoked)	0.845	0.95 (0.54–1.67)		
Nutritional status: malnourished at baseline (v not malnourished at baseline)	0.441	0.57 (0.14–2.37)		
Hypertension (v no hypertension)	0.143	1.58 (0.86–2.90)		
Diabetes mellitus (v no diabetes mellitus)	0.797	1.14 (0.41–3.18)		
FIGO stage III/IV (v stage I/II)	0.004	2.28 (1.30–4.02)	0.006	2.20 (1.25–3.88)
Histology: adeno(squamous) (v squamous cell)	0.163	1.64 (0.82–3.29)		
Overall treatment time ^1^: >50 (v ≤ 50 days)	0.542	1.25 (0.61–2.59)		
Nodal status: N1 (v N0)	0.021	1.99 (1.11–3.59)	0.031	1.91 (1.06–3.45)
Treatment subgroup (v MRI+needles)	0.101		NS	
CT	0.041	1.98 (1.03–3.80)		
MRI	0.881	1.06 (0.51–2.17)		
Disease-free survival				
WHO PS: PS 1–4 (v PS 0)	0.424	1.15 (0.81–1.63)		
Smoking: current or former smoker (v never smoked)	0.397	0.86 (0.61–1.22)		
Nutritional status: malnourished at baseline (v not malnourished at baseline)	0.571	0.80 (0.37–1.72)		
Hypertension (v no hypertension)	0.459	1.16 (0.78–1.72)		
Diabetes mellitus (v no diabetes mellitus)	0.754	0.90 (0.46–1.77)		
FIGO stage III/IV (v stage I/II)	<0.001	2.21 (1.56–3.12)	<0.001	2.13 (1.50–3.03)
Histology: adeno(squamous) (v squamous cell)	<0.001	2.23 (1.49–3.32)	<0.001	2.83 (1.88–4.26)
Nodal status: N1 (v N0)	<0.001	1.94 (1.36–2.76)	<0.001	2.13 (1.49–3.05)
Overall treatment time ^1^: >50 (v ≤ 50 days)	0.017	1.65 (1.09–2.50)	NS	
Treatment subgroup (v MRI+needles)	0.005		0.003	
CT	0.001	2.00 (1.32–3.04)	<0.001	2.09 (1.37–3.19)
MRI	0.071	1.46 (0.97–2.21)	0.105	1.41 (0.93–2.15)
Overall survival				
Age ^2^	<0.001	1.03 (1.02–1.04)	<0.001	1.03 (1.02–1.04)
WHO PS: PS 1–4 (v PS 0)	0.011	1.50 (1.10–2.05)	NS	
Smoking: current or former smoker (vs. never smoked)	0.113	0.78 (0.57–1.06)		
Nutritional status: malnourished at baseline (v not malnourished at baseline)	0.832	0.93 (0.50–1.74)		
Hypertension (v no hypertension)	<0.001	1.81 (1.30–2.53)	NS	
Diabetes mellitus (v no diabetes mellitus)	0.089	1.55 (0.93–2.56)	NS	
FIGO stage III/IV (v stage I/II)	<0.001	2.26 (1.65–3.10)	<0.001	2.11 (1.53–2.90)
Histology: adeno (squamous) (v squamous cell)	0.017	1.62 (1.09–2.42)	0.002	1.87 (1.25–2.81)
Nodal status: N1 (v N0)	0.074	1.33 (0.97–1.82)	<0.001	1.80 (1.28–2.50)
Overall treatment time ^1^: >50 (v ≤ 50 days)	0.099	1.38 (0.94–2.03)	NS	
Treatment subgroup (v MRI+needles)	<0.001		0.015	
CT	0.001	1.92 (1.29–2.86)	0.010	1.71 (1.14–2.57)
MRI	<0.001	1.98 (1.36–2.88)	0.014	1.63 (1.10–2.40)

Bold: *p*-value < 0.05. ^1^ Overall treatment time = time in days from the start of EBRT to the day after the last application of brachytherapy. ^2^ Age = age at diagnosis in years (continuous). HR = hazard ratio; CI = confidence interval; NA = not applicable, NS = not significant; WHO PS = World Health Organization, performance status; FIGO = the International Federation of Gynaecology and Obstetrics; N1 = with lymph node metastases; N0 = no lymph node metastases.

**Table 3:** Severe late toxicity type per treatment group.

		CT		MRI		MRI+Needles		Entire Cohort	
Toxicity Type		n = 75	(%)	n = 103	(%)	n = 219	(%)	n = 397	(%)
Gastro-intestinal		9	(12)	13	(13)	19	(9)	41	(10)
Vaginal		8	(11)	10	(10)	20	(9)	38	(10)
Genitourinary		5	(7)	10	(10)	4	(2)	19	(5)
Other		4	(5)	2	(2)	6	(3)	12	(3)
Overall *	events	26		35		49		110	
	patients	20	(27)	27	(26)	44	(20)	91	(23)

The crude rate is reported per event per toxicity type. The overall toxicity rate is reported both per event and per patient. Legend: * gastrointestinal, vaginal, genitourinary, and/or other severe toxicity.

## Data Availability

Research data are not available at this time. Please contact a corresponding author for any inquiries regarding data sharing in the future.

## Supplementary Materials

The following supporting information can be downloaded at: https://www.mdpi.com/article/10.3390/curroncol30040326/s1, Figure S1: (A) Kaplan-Meier probability estimates for local control stratified per treatment era (CT, MRI, and MRI+needles group) for FIGO stage I/II and FIGO stage III/IV; (B): Kaplan-Meier probability estimates for overall survival stratified per treatment group (CT, MRI, and MRI+needles group) for FIGO stage I/II and FIGO stage III/IV.
