# Supplementary material for: The Effect of Contemporary Brachytherapy Practices on Prognosis in Women with Locally Advanced Cervical Cancer
Source: Curr Oncol. 2023 Apr 19;30(4):4275–88. doi: 10.3390/curroncol30040326 (PMC10137238; doi:10.3390/curroncol30040326)
Supplement: Supplementary file 1 [file curroncol-30-00326-s001.zip › curroncol-2257071-supplementary.pdf]

Article

The Effect of Contemporary Brachytherapy Practices on Prognosis in Women with Locally Advanced Cervical Cancer

Supplementary Materials

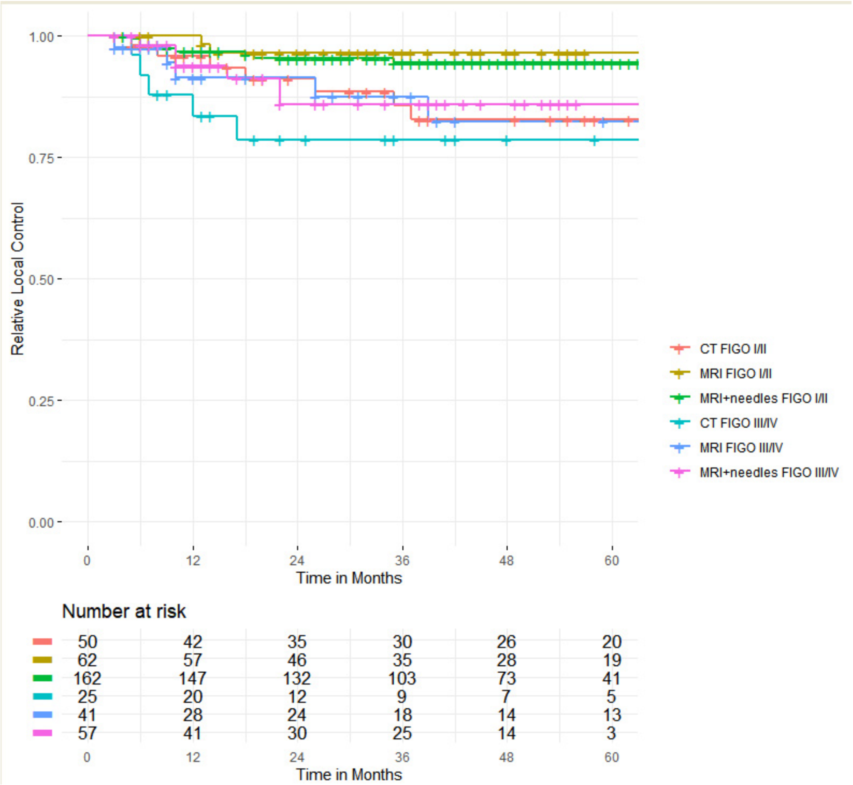

A

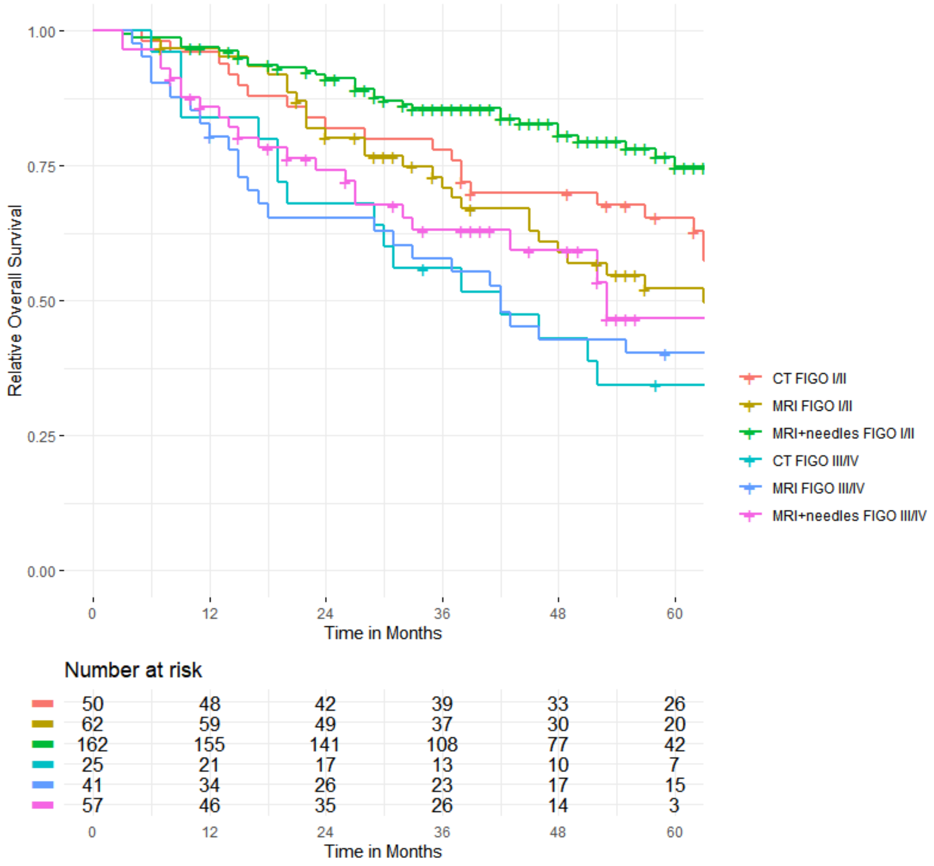

B

Figure S1. (**A**) Kaplan-Meier probability estimates for local control stratified per treatment era (CT, MRI, and MRI+needles group) for FIGO stage I/II and FIGO stage III/IV; (**B**): Kaplan-Meier probability estimates for overall survival stratified per treatment group (CT, MRI, and MRI+needles group) for FIGO stage I/II and FIGO stage III/IV.
